# Guinea pig meat production in South America: Reviewing existing practices, welfare challenges, and opportunities

**DOI:** 10.1017/awf.2025.26

**Published:** 2025-05-05

**Authors:** Gustavo Donoso, Juan Sebastián Galecio, Oscar Giovanny Fuentes-Quisaguano, Monique Pairis-Garcia

**Affiliations:** 1 North Carolina State University, Global Production Animal Welfare, Department of Population Health and Pathobiology, Raleigh, NC, USA; 2 Escuela de Medicina Veterinaria, Universidad San Francisco de Quito USFQ, Quito, Ecuador; 3 Instituto Superior Tecnológico Superarse, Escuela de Veterinaria, Sangolqui- Ecuador

**Keywords:** Animal husbandry, animal welfare, cavy production, Five Domains, food animals, Latin America, traditional livestock

## Abstract

Guinea pigs (*Cavia porcellus*) have been consumed and revered in South American countries since precolonial times and continue to serve as both an important protein source and an economic driver for underserved and remote communities in the region. However, currently, there is limited peer-reviewed research on the welfare status of these animals in meat production systems. This scoping review seeks to provide an overview of guinea pig meat production in the region, highlighting potential welfare challenges and exploring opportunities to advance animal welfare practices within these systems.

## Introduction

Guinea pigs (*Cavia porcellus*) are used in a variety of settings with the majority serving a role as pets or models for laboratory research (Fitria *et al.* 2022). However, in South American countries, guinea pigs are produced as a food source (Dunnum & Salazar-Bravo 2010) and have been raised and consumed in the Andean region since as early as the 15th century. The guinea pig represents an honoured animal in Andean communities, as these animals not only serve as a primary food source but also have a profound spiritual value to the community (Morales 1994).

Producers have an ethical obligation to optimise welfare by eliminating experiences that result in negative affective states for guinea pigs. This focus on animal welfare is not only beneficial to the animal but can also have a significant impact on meat quality, safety and yield (Paranhos da Costa *et al.* 2012). In turn, advancing guinea pig welfare on-farm will not only improve the welfare and performance of the animal itself, but will continue to help maintain a strong social licence and trust between guinea pig producers and consumers (Vargas-Bello-Pérez *et al.* 2017; Alonso *et al.* 2020).

To the authors’ knowledge, there is currently limited peer-reviewed research addressing the welfare of guinea pigs destined for meat production. It is estimated that less than one percent of current animal welfare research in Latin America has focused on this species (Gallo *et al.* 2022). Current literature, to date, has focused primarily on the welfare of guinea pigs as companion (Harrup & Rooney 2020; Wills 2020), laboratory (Mähler *et al.* 2014) or zoo animals (Powell *et al.* 2020). Therefore, this scoping review aims to provide an overview of meat guinea pig production and welfare categorised generally within the framework of the Five Domains (Mellor *et al.* 2020) and highlights future opportunities for animal welfare advancement in guinea pig production systems.

## Industry overview

### Current production statistics

Peru, Ecuador, Bolivia and Colombia are the biggest producers and consumers of guinea pig meat. In 2021, it was estimated that Peru produced over 25 million guinea pigs with a local consumption around 22,000 tons (Ministerio de Desarrollo Agrario y Riego 2023). The Ecuadorian Ministry of Agriculture (2019) reported that at least 21 million guinea pigs were consumed in the country, although no information was provided regarding how this number was estimated. This estimate places Ecuador as the second largest producer in Latin America.

On the other hand, Bolivia and Colombia are considered smaller but still relevant producers of guinea pig meat, with both countries estimated to have inventories of at least 6 million and 2.8 million guinea pigs, respectively (Forero *et al.* 2023; Pinchao-Pinchao *et al.* 2024). However, current information on true inventory numbers is lacking at present and further work is needed to quantify guinea pig production across countries.

### Production systems

Guinea pig meat production can be categorised into three production types: family owned and operated (family-type), family owned and commercially operated (family-commercial) and commercial owned and operated (commercial-type). Family-type production systems are the most common in the Andes and management and care of animals is the primary responsibility of women in rural communities (Lammers *et al.* 2009; Sánchez-Macías *et al.* 2018).

Record-keeping is a crucial aspect of any production system, regardless of its size. However, there is a noticeable lack of peer-reviewed studies specifically addressing record-keeping practices in guinea pig meat production in the scientific literature. Only a local study supported by the National Institute of Agricultural Research (INIAP) was found, which reported that 88% of the interviewed guinea pig producers in Ecuador do not maintain written records of production parameters (Camacho & Patiño 2022).

This lack of documentation may pose challenges for safeguarding animal welfare if veterinarians and producers are unable to effectively track and monitor population health and/or disease risk. Record-keeping has been recognised as an important management tool for improving daily farm operations (Mwanga *et al.* 2020) and can be easily trained via workshops and outreach targeted for producers (Tang Dalsgaard *et al.* 2005). However, long-term behavioural change is needed to ensure this activity is maintained (Tham-Agyekum *et al.* 2010).

#### Breeding and genetic selection

There is a debate in the literature regarding whether guinea pigs originated from *Cavia tschudii* or *Cavia aperea.* More recent genomic studies have suggested that guinea pigs likely originated from *C. tschudii*, which was later domesticated into the pre-Columbian species *C. porcellus*, commonly known as ‘Criollo’, a breed still widely used for meat production today (Spotorno *et al.* 2006; Walker *et al.* 2014; Kaiser *et al.* 2015). Guinea pigs exhibit early reproductive maturity, with females reaching sexual maturity at about one month of age, having a gestation period of 63–72 days, and producing litters of 1–7 pups, while males reach sexual maturity at 2–3 months of age (Kaiser et al. 2024).

**Figure 1.**
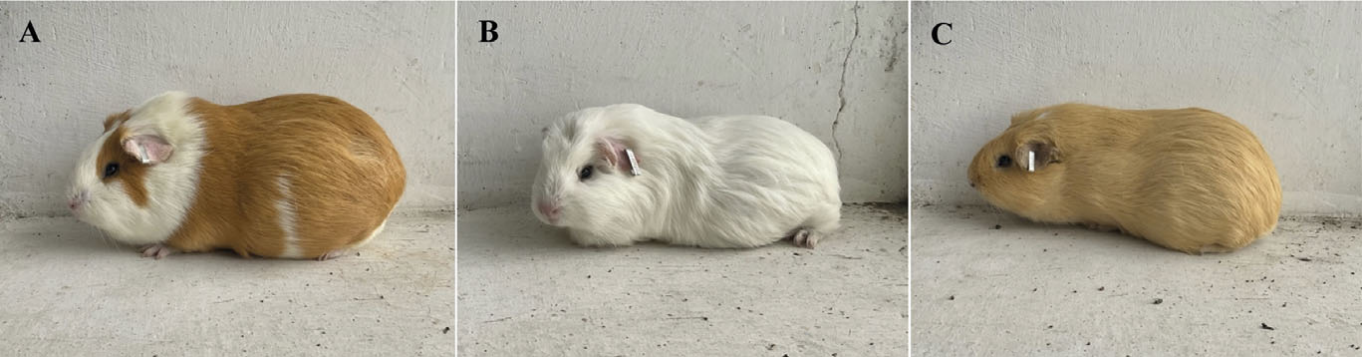
Common guinea pig (*Cavia porcellus*) breeds used for meat production showing (A) the Peru breed, characterised by its bi- or tri-colour pattern coat composed primarily of orange and white, (B) the Andina breed characterised by a full body white coat and (C) the Inti breed characterised by a full body golden or yellow coat.

Genetic selection programmes initiated in the 1970s by the Peruvian Institute for Agriculture Innovation (INIAA) began selecting more commercially relevant breeds from the ‘Criollo’ as a means of providing a solution for food-insecure communities within the Andean region (Chauca 1995). These breeds are larger and heavier, resulting in a more efficient meat yield per animal (Lammers *et al.* 2009; Sánchez-Macías *et al.* 2018). The three main breeds used today are the Perú, Andina, and Inti breeds (Figure 1; Chauca 2007).

## Behavioural interactions

In the wild, rodents of the genus *Cavia* live in groups of 5–10 animals and have a dominant male guinea pig within the group (Hargaden & Singer 2012). In contrast to other members of the rodent family (i.e. mice and rats), guinea pigs are diurnal (Sirois 2005). Domestic guinea pigs share many common behavioural features with their wild counterparts (*C. aperea*) but differ in social and bonding aspects and do not have a strong motivation to explore their surrounding environment (Kaiser *et al.* 2015). Guinea pigs exhibit thigmotactic behaviour, a trait developed from their ancestral habit of residing in dense undergrowth adjacent to open foraging areas to reduce predation risk, which highlights the biological importance of environmental enrichment that provides refuge or cover for this species (Asher *et al.* 2004; Adrian & Sachser 2011; Byrd *et al.* 2016).

As a small prey species, guinea pigs are predisposed to stress associated with novel or fearful situations (McBride 2017). In response to stress, guinea pigs tend to experience tonic immobility, an innate response of profound inactivity and relative lack of responsiveness to the environment, which is also known as reflex immobility, animal hypnosis, ‘playing dead’, or fear paralysis (da Silva & Menescal-de-Oliveira 2006).

Tonic immobility can be elicited in many fearful situations or by strong tactile stimuli, which include inappropriate handling or excessive noise in the environment. This stress response can be reduced by training caretakers on proper handling, restraint techniques and using slow, quiet behaviour when working with and handling guinea pigs (Rocha *et al.* 2017). Humane handling of production guinea pigs could confer multiple benefits by reducing stress associated with this activity as well as having positive effects on reproductive performance (Klaus *et al.* 2013; Sánchez-Macías *et al*. 2016).

In addition to a pronounced stress response, guinea pigs can also display aggressive behaviour among their littermates, although they are less aggressive than their wild counterpart (*C. aperea*) since newer breeds have been genetically selected to be more docile (Künzl *et al.* 2003; Brust & Guenther 2017). Aggression is most commonly observed between sexually mature, intact males (Harper 1976; Mínguez Balaguer *et al.* 2019). In production systems, a harem-type system is employed for reproduction, where a single male is housed with 10 to 12 females (Usca *et al.* 2022) resulting in few aggressive interactions within the group.

When housed in large populations with multiple males (10–15 or more) during the growing period (45–60 days), animals will form subunits whereby an alpha male dominates over subordinate males (Verzola-Olivio *et al.* 2021). The management of colonies during adolescence plays a crucial role in shaping social behaviour, as males that have not been raised in large mixed-sex colonies exhibit more aggressive behaviours toward other males due to the lack of social acclimation during this development period (Sachser *et al.* 2011a). In production settings, mixing males of unknown origin or those without prior colony exposure can lead to frequent agonistic interactions. For welfare reasons, such practices should be avoided.

Stable social groups are not only important for reducing agonistic behaviours but also have a significant impact on reproductive behaviour. Female guinea pigs exposed to social instability during pregnancy give birth to boars characterised by less pronounced masculine traits (behavioural infantilisation) and masculinised female progeny resulting in long-term impacts on reproduction (Sachser *et al.* 2011b).

## Environment

### Housing

In family-type production systems, guinea pigs are primarily raised in the kitchen and support the nutritional needs of the family (Avilés *et al.* 2014). Guinea pigs are also commonly kept in small cages near the main building or other parts of the house and will be raised for consumption at around three months. A single-family household will typically care for ten to 15 guinea pigs, thereby enabling the family to control access to and frequency of their most readily available protein source.

Despite being the most common production system in South America, very little is known about the current welfare state of guinea pigs living in family-type production systems. Future efforts need not only to address the welfare of the animal but also that of those raising guinea pigs, given the significant threat of zoonotic diseases associated with such close contact (Vasco *et al.* 2016; Lowenstein *et al.* 2020).

In contrast to family-type production, family-commercial and commercial production systems house guinea pigs in either wire-mesh cages elevated from the floor or rectangular bedded pens enclosed with either cement or bricks (Rico & Rivas 2003). Although there are no science-based guidelines specific to stocking density regarding wire-mesh cages, the standard is 1 m × 1 m and typically hold 10–15 guinea pigs. In solid flooring rectangular pen systems, stocking recommendations are provided and can be found in Table 1.

**Table 1.** Stocking densities for rectangular pens in guinea pig (Cavia porcellus) productions

Production stages	Stocking density (m^2^)
Growing males	0.16
Growing females	0.14
Fattening males	0.24
Fattening females	0.18
Breeding	0.28

* Summarised from Cáceres *et al.* (2004).

For the latter system, bedding is important but has not been thoroughly studied in production settings. In laboratory facilities, bedding (~7 cm depth) is commonly placed in pens as an absorbent material and is composed primarily of small grain straw or rice hulls, that also serves as an enrichment to allow guinea pigs to express species-specific behaviours such as burrowing and hiding (Scharmann 1991). Guinea pigs will maintain distinct areas for sleeping, eating and defaecating within the bedding area (Lammers *et al.* 2009). However, quantity and quality of space should still be explored, emphasising the need for further research into housing conditions in farms to ensure optimal welfare for this species.

A study that compared guinea pigs housed on wire floors and rectangular bedded pens, demonstrated no differences in performance (i.e. growth, feed intake, carcase traits, mortality; Mínguez Balaguer *et al.* 2019). However, potential welfare issues may still arise due to increased risk of injury or disease due to flooring. Animals housed on wire cages are more likely to develop feet and leg issues, including pressure sores, pododermatitis, and/or fractures (Fawcett 2011). Studies evaluating welfare challenges associated with wire flooring in other species, such as rabbits and chickens, have demonstrated that wire cages increase pododermatitis risk (Rosell & de la Fuente 2009), osteoporosis and fractures (Regmi *et al.* 2016).

This evidence suggests that guinea pig welfare may still be compromised when using wire flooring which could lead potentially to development of foot or leg problems, resulting in the negative affective state of pain even in subclinical or mild cases. More work is needed to assess conditions that may lead to compromised welfare, including broken or poorly maintained cages and inappropriately managed litter with high moisture levels.

### Handling and transportation

**Figure 2.**
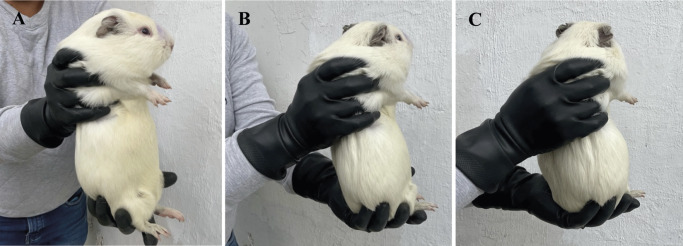
Handling of a medium-sized, meat production guinea pig (*Cavia porcellus*) showing (A) side view, (B) ¾ view and (C) back view.

Correct handling and restraint are critical components for ensuring optimal animal welfare in any farm animal. Fear-free handling in laboratory settings can be achieved by gently picking up the animal with both hands, positioning one hand to support the shoulders and chest of the guinea pig while the other hand supports the hindquarters (Figure 2). The guinea pig should be firmly secured against the handler’s body, which is particularly important for pregnant guinea pigs (Kaiser *et al.* 2024). Animals should never be grabbed or lifted by the skin on the scruff of the neck, as this can cause stress and discomfort (Zimmerman *et al.* 2015).

Even though this method works well for animals kept as pets or in laboratory settings and can potentially work for small to medium size guinea pigs in production, it is the authors’ experience that the heavy weight of meat guinea pigs (1,000–1,200 g) can make this handling technique challenging. More studies are needed regarding the correct handling of larger animals in production settings.

Currently, there is a lack of studies regarding transportation for guinea pigs in production and no official data were found concerning their transportation in the main producing countries. This is critical, as transportation experience has been shown to induce a marked psychological and physical stress response in guinea pigs and could be compounded if vehicle movement is rough and feed and water withdrawal is prolonged (Fisher *et al.* 2009).

Transportation in laboratory guinea pigs has been shown to induce an increased cortisol response (Walters *et al.* 2012) and weight loss has also been reported (Stemkens-Sevens *et al.* 2009). For guinea pigs, fasting is generally recommended for solids, liquids or both at 12–14 h prior to slaughter (Kouakou *et al.* 2013; Sánchez-Macías *et al.* 2016), however, no scientific basis was found for these recommendations and further studies are needed.

## Nutrition

Feed is the primary expense in guinea pig production, representing 44.2% of total costs (Pascual *et al.* 2017). This economic importance has made nutrition one of the most studied aspects of guinea pig rearing and nutrient requirements have been thoroughly described (National Research Council 1995).

Nutrition varies depending on the production type. In family-type systems, females and males are not separated and are fed together with discarded food or vegetables derived from kitchen waste or agricultural waste products (Sánchez-Macías *et al.* 2018). Inconsistent feedings with poor quality feedstuffs may result in aggression associated with resource competition, particularly in intact males and such behaviours may result in injury to the guinea pig, potentially impacting carcase quality (e.g. scarring; Mínguez Balaguer *et al.* 2019).

On the other hand, family-commercial and commercial-type production systems use formulated concentrate diets along with grass (Chirinos-Peinado *et al.* 2024). Feed texture is also an important component of guinea pig nutrition as guinea pigs require abrasive feedstuffs to help wear down continuously growing teeth to prevent malocclusion or other teeth problems (Müller *et al.* 2015; Witkowska *et al.* 2016). Lastly, guinea pig diets must be supplemented with vitamin C (ascorbic acid) as this species are unable to synthesise it themselves and reduced levels can lead to potential health issues, including scurvy (Witkowska *et al.* 2016).

## Health

Human and animal health and welfare are closely interlinked because ensuring that animals are healthy and well cared for provides a safe protein source for people (Schneider & Tarawali 2021). Guinea pigs are susceptible to multiple diseases, including dental and skin problems (Minarikova *et al.* 2015). On-farm, skin diseases caused by external parasites are a persistent problem with previous work demonstrating prevalence to be as high as 67% of the population (Santos *et al.* 2020). By controlling the environment, this problem could be solved as guinea pigs raised in crowded and uncontrolled environments have major incidence of ectoparasites, diminished bodyweight and altered haematological parameters (Fitria *et al.* 2022).

Infectious diseases also play a major role in guinea pig production systems and are not just an animal welfare issue but also a potential source of zoonoses for those caring for the animals. The principal disease affecting guinea pig production is *Salmonella* spp (Sánchez-Macías *et al.* 2018) and is the cause of high mortality and morbidity (Carhuaricra Huaman *et al.* 2022). However, guinea pigs can also serve as subclinical carriers and transmit disease to other animals and humans (Fournier *et al.* 2015). Diagnosis and control of this disease is of critical importance for both animal welfare and public health.

One major challenge for the delicate equilibrium that exists between health and welfare is the use of antibiotics and antimicrobial resistance (AMR). AMR has been on the rise globally (Kasimanickam *et al.* 2021) and a major contributor to this is the use of antibiotics on animals, not only as a tool for treatment, but also as a means of improving productivity (Bengtsson & Greko 2014).

A qualitative study conducted in Ecuador found producers believe that antibiotics are included in commercial animal feed given to their production animals and that most reacted positively to this idea, due to the belief that it would help their animals grow faster and fight ‘parasites’. In addition to antibiotics in the feed, producers also reported treating animals with additional ‘remedies’ they had in their possession at home (Lowenstein *et al.* 2016).

Another study surveying small producers in Peru found knowledge of antimicrobials to be linked to farmers’ educational level, monthly income, knowledge of the animal health authority and farm features (Benavides *et al.* 2021). Authors noted that farmers reported using antibiotics for many diseases. While most of the treatments were prescribed by veterinarians, 29% reported acquiring the antibiotics from a ‘veterinary store’ and 19% also said that they could have acquired them either from the veterinarian or by the ‘veterinary store’.

This ready access to medications could severely impact the welfare of animals and pose a direct risk for AMR build-up in Latin America and beyond. Even though this survey has shown access to a veterinarian for this species to be feasible in certain provinces of Peru, there has been no research conducted regarding access to veterinary care for small or rural communities in other parts of Peru or in other countries.

## Mental state

### Pain

Pain is a complex experience with sensory and emotional components, and its recognition in food-producing animals has become increasingly important due to the growing awareness of animal welfare (Reid *et al.* 2013). Around the world, people are demanding pain management as an important part of safeguarding food animal welfare and maintaining trust with society (Steagall *et al.* 2021).

Pain has been studied in guinea pigs and behaviours associated with this experience have been described as an indicator for its assessment (Ellen *et al.* 2016; Benedetti *et al.* 2024). However, the development and validation of studies into pain assessment have been limited to companion (Benedetti *et al.* 2024), or laboratory animals (Dunbar *et al.* 2016; Ellen *et al.* 2016; Oliver *et al.* 2016) and, to date, there have been none published on pain assessment and management in farm animal guinea pig species (Flecknell 2018; Foley *et al.* 2019). Given these animals are raised for consumption, additional work is needed to ensure pharmaceutical products used for pain control have residue data and withdrawal periods established.

Potential sources of pain include, but are not limited to, failure to treat diseases, traumatic injuries, and administration of improper feed that could lead to bloating and gastrointestinal problems (Harkness *et al.* 2007). In meat production, the widespread use of castration for reproductive control and aggression management could represent a significant source of pain, particularly in commercial farms where these practices are more common (Mínguez Balaguer *et al.* 2019).

### Slaughter and euthanasia

Humane slaughter is an important component of food animal welfare. Animals that are slaughtered for meat production should experience minimal stress or suffering associated with the experience (Nakyinsige *et al.* 2013). Cervical dislocation is a commonly used method for guinea pig slaughter in both family and commercial productions in Peru due to its perceived practicality (Mota-Rojas *et al.* 2012). However, research has shown this practice to be ineffective and that its use should be discouraged since it often results in incomplete insensibility (Limon *et al.* 2012, 2016).

In contrast to cervical dislocation, penetrating captive bolt and head electrical stunning are two methods that, when properly implemented, can ensure insensibility and, in the case of penetrating captive bolt, also death (Limon *et al.* 2016). Penetrating captive bolt has been found to be a good method for euthanasia that can achieve good welfare standards in research settings (Cohen *et al.* 2020) and this method could be easily applied on-farm for this purpose too. Electrical stunning, while effective for inducing insensibility, can also impact meat quality by reducing pH levels and decreasing water retention (Mota-Rojas *et al.* 2012).

Additionally, the guidelines from the World Organisation for Animal Health (WOAH) highlight the critical importance of animals being stunned immediately following restraint and prior to slaughter or euthanasia. All stunning and euthanasia processes should be followed by proper cutting and bleeding to ensure complete exsanguination. Adhering to these practices is essential for maintaining high standards of animal welfare, health safety, and meat quality (World Organisation for Animal Health 2024).

## Other current welfare issues

### Preparedness of future generations

Future generations are important for maintaining and improving animal welfare in the region. Countries that produce guinea pigs are members of the WOAH, and this institution has proposed the inclusion of animal welfare in the curriculum of universities for all of its members (Bui & Anh 2013). By 2008, 63% of the veterinary schools in Latin America had at least one animal welfare course, while by 2016 of 100 veterinary schools surveyed in the region the number increased to 98%, however there was no agreement of issues to be addressed or the depth of the course content (Gallo *et al.* 2010, 2022; Tadich *et al.* 2010).

Attempts at formulating unified content that could form the basis for animal welfare courses in Latin America have been published (Mota-Rojas *et al.* 2018), although these represent a welcome addition to the curriculum, none address the behaviour or welfare of the guinea pig. The importance of this animal to the region (in particular the Andes) makes incorporation of this topic highly important.

### One Welfare and food security

One Welfare is a framework that recognises the interconnections between the welfare of people, animals and the environment (Garcia-Pinillos 2018). Guinea pigs have been recognised as an important source of income to reduce food insecurity in socially deprived communities in South America and as a way of empowering rural woman with financial agency, given they serve as the primary caretaker role in guinea pig farms (Lammers *et al.* 2009).

Recognising the connection between human and animal welfare, it is essential to consider the producer’s well-being and motivation in driving improvements in animal welfare. Producers often face significant social and economic pressures that can influence how they care for their animals (Garcia-Pinillos & Huertas Canén 2023). If we aim to improve the welfare of guinea pigs in meat-producing countries, we must recognise the needs and perspectives of producers whose livelihoods depend on guinea pig production. Supporting governments and producers in developing policies that safeguard animal welfare is essential. However, these policies must be designed with respect for the cultural realities of each region, rather than forcing practices that may have worked elsewhere (Marchant *et al.* 2023; Galindo *et al.* 2024).

## Animal welfare implications and future directions

As highlighted by this review, many challenges remain regarding the welfare of meat-production guinea pigs. Further scientific studies are needed to address the issues identified in this review and strive for the improvement of guinea pig welfare in the region, as only a few papers were found when trying to address potential welfare issues for these animals in this setting. This may be because most journals use English as their language of choice, while all the guinea pig producing countries in South America have Spanish as their national language, an issue highlighted previously (Diana *et al.* 2021; Pasteur *et al.* 2024).

However, most Spanish publications found during the present review were not annexed to journals and consequently did not undergo the peer review process for publication. Keeping in mind that the use of scientific literature has been crucial when identifying critical points for the welfare of farm animals in the past and has guided the progress of education and legislation (Gallo *et al.* 2022), sound scientific evidence should be a top priority for producing countries. Encouraging local governments, research institutions, farmers and animal professionals to aim for the responsible production of this species based on sound scientific evidence and through a multidisciplinary lens such as the One Welfare scheme (Garcia-Pinillos 2018) can provide improvement of local economy of underserved groups of society, well-being of both animals and people and, in general, achieve gains in development for the regions that produce this animal as a protein source.
